# Impact of Sugar Beet Pulp-Based Diets on the Chemical Composition, Mineral Profile, and Chitin Yield of Black Soldier Fly (*Hermetia illucens* L.) Larvae

**DOI:** 10.3390/ani16213380

**Published:** 2026-10-27

**Authors:** Nerijus Naglius, Povilas Mulerčikas, Aurelija Ramanauskaitė, Irmantas Parasotas, Gintarė Naglė

**Affiliations:** 1 Institute of Agricultural Sciences, Faculty of Agronomy, Vytautas Magnus University Agriculture Academy, K. Donelaičio g. 58, 44248 Kaunas, Lithuania; 2 Institute of Agriculture, Lithuanian Research Centre for Agriculture and Forestry, Instituto al. 1, Akademija, 58344 Kėdainiai, Lithuania; 3 Independent Researcher, 11342 Vilnius, Lithuania

**Keywords:** *Hermetia illucens*, sugar beet pulp, molasses, *Trichoderma* sp., proximate composition, crude protein, crude ash, mineral profile, calcium, phosphorus, extracted chitin yield

## Abstract

Utilizing agro-industrial by-products such as sugar beet pulp (SBP) in black soldier fly (*Hermetia illucens* L.) rearing is an attractive strategy for circular feed production; however, its consequences on larval chemical composition and cuticular mineralization require detailed characterization. This study evaluated the effects of SBP inclusion, with or without *Trichoderma* sp. inoculation or beet molasses addition, on the proximate composition, macro- and trace mineral profiles, and gravimetric chitin yield of whole *H. illucens* larvae. Five-day-old larvae were reared across three independent biological cycles (*n* = 3 rearing containers per treatment) on four diets: Control (100% wheat bran), Option 1 (60% wheat bran + 40% SBP), Option 2 (60% wheat bran + 40% SBP with *Trichoderma* sp. at 1 × 10^6^ CFU/g), and Option 3 (60% wheat bran + 30% SBP + 10% molasses). Larvae were harvested without a pre-harvest fasting period to reflect commercial whole-larva processing. Dietary inclusion of SBP significantly decreased larval crude protein (44.66 ± 2.88% DM in Option 1, 44.36 ± 3.12% DM in Option 2, and 45.25 ± 0.97% DM in Option 3 vs. 50.85 ± 3.23% DM in Control; *p* = 0.038), reflecting the lower protein density of SBP. Crude fat (32.18–36.32% DM; *p* = 0.774) and water-soluble carbohydrates (1.80–2.20% DM; *p* = 0.388) did not differ significantly among treatments. SBP inclusion markedly increased the crude ash content (10.59–13.68% DM vs. 5.75 ± 0.52% DM in Control; *p* < 0.001) and whole-body calcium (7.46–8.84% DM vs. 2.43 ± 1.39% DM in Control; *p* = 0.003), driving the larval Ca:P ratio from 2.45:1 to 8.04:1. Manganese concentrations were elevated in all SBP diets (661.2–700.6 mg/kg vs. 589.9 ± 71.7 mg/kg in Control; *p* = 0.041), while iron was significantly higher in Option 1 (286.9 ± 71.5 mg/kg vs. 168.9 ± 34.4 mg/kg in Control; *p* = 0.024). The gravimetric chitin yield was significantly higher on 40% SBP diets (7.75 ± 0.22% DM in Option 1; 7.82 ± 0.18% DM in Option 2) than in the Control (7.34 ± 0.15% DM; *p* = 0.004), whereas Option 3 yielded the lowest chitin content (7.14 ± 0.12% DM). Fungal inoculation produced no significant differences over SBP alone. These results demonstrate that SBP-based diets substantially increase whole-body ash and calcium levels while altering chitin yields, providing critical compositional benchmarks for tailoring insect meal to non-ruminant livestock feeding.

## Simple Summary

Utilizing agro-industrial by-products like sugar beet pulp (SBP) in black soldier fly (*Hermetia illucens*) farming can enhance circular feed sustainability, yet its effects on larval nutrition and mineral accumulation remain poorly understood. In this study, we evaluated the effects of replacing wheat bran with SBP (with or without *Trichoderma* sp. or beet molasses) on the nutritional value, mineral composition, and chitin yield of the larvae. The results showed that diets containing sugar beet pulp reduced the crude protein content due to nutritional dilution, but crude fat levels remained stable, demonstrating robust larval energy metabolism. Crucially, SBP diets dramatically increased crude ash and calcium levels—widening the calcium-to-phosphorus ratio up to 8:1—and moderately altered chitin deposition. These findings provide critical benchmarks for feed manufacturers, indicating that SBP-reared insect meal is an excellent natural calcium and energy supplement for laying poultry, though it requires mineral rebalancing when formulated for broilers or young pigs.

## 1. Introduction

Valorizing agro-industrial side-streams through insect bioconversion provides a viable pathway to achieve circular economy goals and sustainable protein production [1,2]. Conventional protein commodities for livestock and aquaculture diets, notably soybean meal and fishmeal, are associated with substantial environmental challenges, including land-use change, deforestation, and marine resource overexploitation [1,2]. The black soldier fly, *Hermetia illucens* (L.) (Diptera: Stratiomyidae), has emerged as an exceptionally efficient bio-converter capable of transforming low-value agro-industrial by-products into high-value larval biomass rich in proteins and lipids [3,4,5].

The physiological plasticity of *H. illucens* larvae allows them to thrive on heterogeneous, high-moisture, and moderately fibrous substrates that are unsuitable for other mass-reared insects like yellow mealworms (*Tenebrio molitor*) or house crickets (*Acheta domesticus*) [4,6]. While *T. molitor* and crickets typically exhibit inverted calcium-to-phosphorus (Ca:P) ratios ranging from 0.1:1 to 0.3:1 [7], *H. illucens* larvae uniquely mineralize their cuticular exoskeleton with calcium carbonate, establishing baseline Ca:P ratios of 2:1 to 4:1 even on cereal diets [7,8]. Although larvae lack potent endogenous cellulases, their symbiotic gut microbiome—comprising cellulolytic and pectinolytic bacterial taxa—plays an integral role in fermenting complex plant polysaccharides [9,10,11].

The proximate composition of *H. illucens* is known to respond dynamically to rearing substrates [9,12,13]. On a dry matter (DM) basis, the crude protein content generally varies between 35% and 52%, providing a favorable essential amino acid balance [12,14]. Crude fat levels vary widely between 15% and 40% DM, depending on the substrate nutrient density and harvest instar, accumulating rapidly during the fifth and sixth larval stages [15,16]. Simultaneously, cuticular development and mineral deposition result in crude ash contents ranging between 5% and 15% DM [8,17]. Chitin, a structural nitrogenous polymer (*β*-(1,4)-poly-*N*-acetyl-D-glucosamine) tightly integrated with cuticular proteins and mineral precipitates, constitutes approximately 4% to 9% of larval dry weight [18,19,20,21].

In the European Union, processed insect proteins (PAPs) derived from *H. illucens* are officially authorized for aquaculture, poultry, and swine feeds under Regulation (EC) No. 1069/2009 and Commission Regulation (EU) 2021/1372 [22,23]. To support industrial feed formulation, substrates must be safe, free of prohibited contaminants [24,25], and consistent in nutrient delivery [26].

Sugar beet pulp (SBP) represents a major agro-industrial side-stream generated during industrial sugar refining, with global production exceeding 50 million metric tons of wet pulp annually [27]. SBP is characterized by high levels of structural carbohydrates (pectin, cellulose, and hemicellulose; >65% DM) and low crude protein contents (7–10% DM) [13,28]. Furthermore, industrial lime carbonation during sucrose extraction enriches residual pulp with calcium precipitates [13,27]. While several studies have explored *H. illucens* growth on fruit pomace, spent grains, and municipal biowaste [3,8,26,29], research examining SBP as a primary dietary component remains limited [13]. Due to the high fiber density and low nitrogen content of SBP, biological interventions such as fungal inoculation or carbohydrate supplementation have been proposed to enhance substrate accessibility [30,31]. Inoculation with *Trichoderma* species, known for their abundant production of cellulases, hemicellulases, and pectinases, can partially hydrolyze recalcitrant plant cell walls, potentially increasing nutrient bioaccessibility for insect larvae [31]. Concurrently, co-supplementation with soluble carbohydrates, such as industrial beet molasses, may provide readily fermentable energy to support larval lipogenesis and metabolic growth [30].

Despite the growing body of insect nutrition literature, significant knowledge gaps persist regarding how high-fiber, calcium-rich SBP matrices affect the whole-body macromineral balance (Ca, P, K, Na, Mg), trace element accumulation (Fe, Zn, Mn, Cu), and gravimetric chitin deposition in *H. illucens*. We hypothesized that replacing wheat bran with SBP would dilute larval crude protein while promoting cuticular mineralization (elevating crude ash and calcium levels) and modulating the chitin yield, whereas *Trichoderma* inoculation or molasses supplementation would alter nutrient availability without impairing larval lipid accretion. Therefore, this study aimed to determine the effects of SBP-based diets, with and without *Trichoderma* sp. inoculation or molasses addition, on the proximate macronutrients, mineral profiles, and gravimetric chitin yields of *H. illucens* larvae.

## 2. Materials and Methods

### 2.1. Larval Origin and Rearing Setup

The experiment was conducted using 5-day-old *Hermetia illucens* larvae obtained directly from the active breeding colony of the UAB Insectum (Kaunas, Lithuania). All larvae originated from the same continuous parent genetic colony across all experimental cycles to eliminate genetic drift. Larvae were harvested 5 days post-hatching from nursery feeding crates and separated from residual feed by manual sieving. The mean initial individual weight was established at 10.0 ± 0.4 mg wet weight (corresponding to 2.8 ± 0.1 mg DM per larva) by weighing and oven-drying representative aliquots at 105 °C to a constant weight.

**Figure 1:**
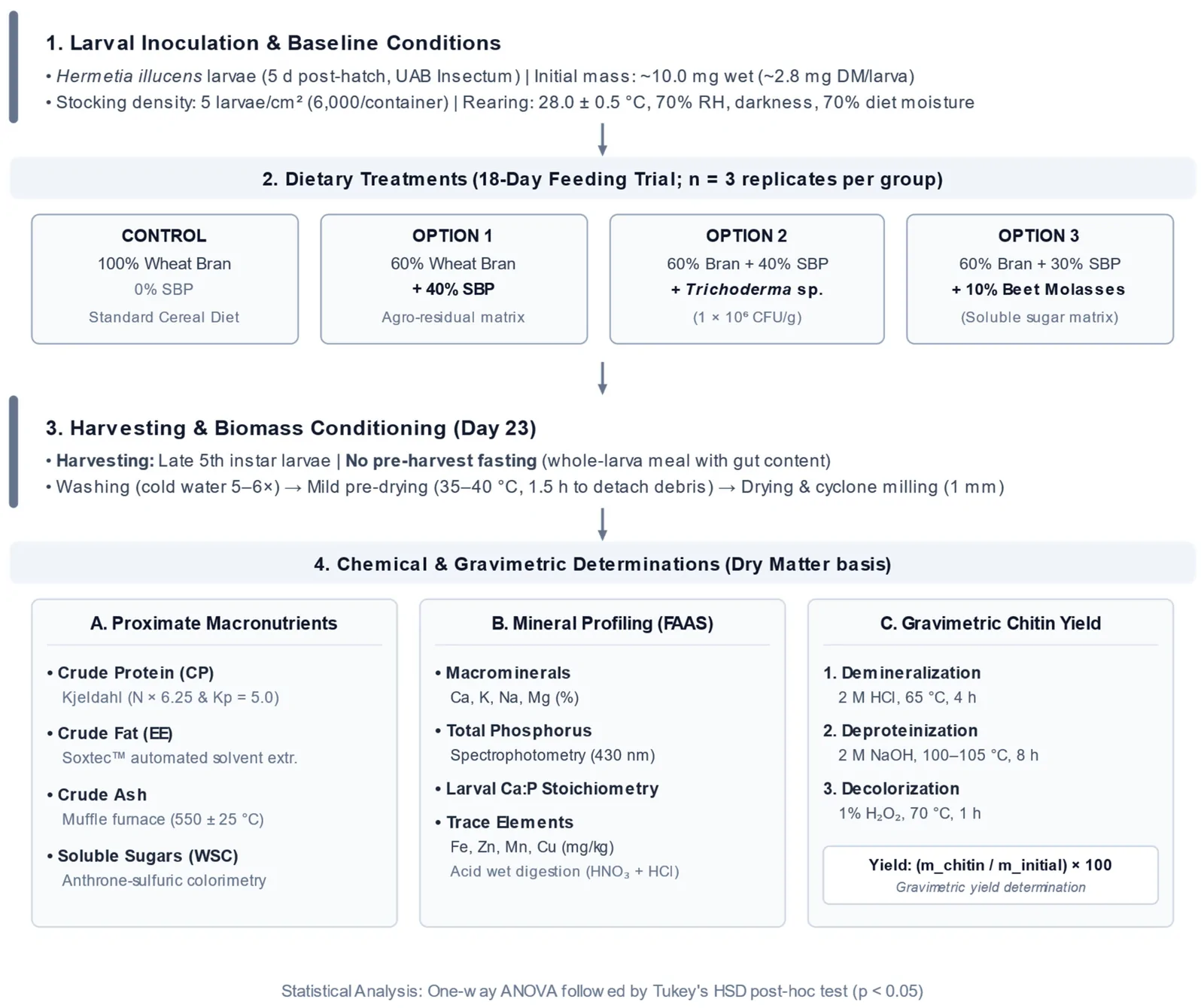
Schematic diagram of the experimental workflow, dietary treatments, and sample processing.

Standardized polypropylene rearing containers (40 × 30 × 22 cm) were seeded at a stocking density of 5 larvae/cm^2^ (6000 larvae per container; total initial wet biomass 60.0 ± 0.5 g per container) [32,33,34]. In accordance with insect bioconversion methodologies [29,34], an experimental container serving as a discrete rearing unit was established with *n* = 3 biological replicates per dietary treatment across sequential rearing cycles (total *N* = 12 container units; Figure 1).

### 2.2. Dietary Treatments and Rearing Conditions

Four dietary formulations were evaluated (Table 1). Fresh SBP and industrial beet molasses were obtained from Nordic Sugar Kėdainiai (Lithuania), and wheat bran was obtained from Kauno Grūdai (Lithuania). The dietary inclusion rate of 40% SBP in Option 1 and Option 2 was selected based on pilot trials and previous research showing that exceeding 40–50% fiber-rich by-products impairs larval intake due to excessive water holding and a high bulk density [13,29]. In Option 3, SBP was reduced to 30% and supplemented with 10% molasses to provide readily available fermentable sugars without creating an excessively dense or wet slurry.

For Option 2, a commercial biological preparation containing viable conidia of *Trichoderma* sp. (*Trichoderma harzianum*/*viride*, Bioenergy LT, Panevėžys, Lithuania; guaranteed spore titer of 1 × 10^9^ CFU/g carrier powder) was thoroughly blended into the dry feed mixture at a target concentration of 1 × 10^6^ CFU/g dry substrate immediately prior to moisture adjustment, without an extended solid-state pre-fermentation period.

All diets were adjusted to a 70.0 ± 1.0% initial moisture content using distilled water. Feeding was administered once at the beginning of the trial at a feeding rate of 0.06 g wet substrate per larva (equivalent to 0.018 g DM per larva; 108.0 g dry feed mass per container) [33,34,35].

Because experimental chemical analysis of the raw ingredient batches was not performed prior to the feeding trials, baseline proximate and mineral compositions of the dietary ingredients were compiled from established feed databases and the peer-reviewed literature [13,27,36,37] to provide a nutritional benchmark (Table S1).

Rearing was conducted in a climate-controlled chamber at 28.0 ± 0.5 °C and 70.0 ± 2.0% relative humidity in complete darkness (24 h dark, except during routine inspection) across three sequential independent biological cycles (Cycle 1: 30 May–16 June 2025; Cycle 2: 28 July–15 August 2025; Cycle 3: 08 September–25 September 2025; 18-day rearing trial per cycle). The experimental unit was the individual rearing container (*n* = 3 container observations per dietary treatment).

### 2.3. Sample Collection and Preparation

Larvae were harvested at day 23 of total development (18 days of feeding trial), when larvae were in the late fifth/early sixth instar prior to entering the non-feeding prepupal stage [21]. In accordance with standard industrial whole-larva meal manufacturing practices, larvae were harvested directly without an intentional 24 h pre-harvest fasting period (gut clearance).

Larvae were separated from residual frass [38] using a 3 mm mechanical sieve, washed under cold running tap water, and gently agitated five to six times to remove adhering substrate particles, followed by a final rinse with deionized water. Washed larvae were blotted on paper towels and subjected to a mild pre-drying step in a forced-air circulation oven at 35–40 °C for 1.5 h. This mild thermal step was applied exclusively to detach any lingering microscopic bran or fiber fragments from the larval cuticle via gentle dry sieving (3 mm mesh). Maintaining temperatures strictly at 35–40 °C prevented thermal enzymatic breakdown, volatile losses, and lipid auto-oxidation prior to final analytical drying.

Cleaned larvae were subsequently dried in a forced-air oven at 65 °C for 24 h and finalized at 105 ± 2 °C for 4 h to a constant weight to determine the dry matter (DM) content in accordance with ISO 6496:1999. Dried larval samples were pulverized using a laboratory cyclone mill (Cyclotec 1093; FOSS, Hillerød, Denmark) through a 1 mm screen and stored in vacuum-sealed plastic bags at 4 °C until chemical analysis.

### 2.4. Chemical Analyses

Chemical analyses were carried out at the Chemical Research Laboratory of the Institute of Agriculture, LAMMC. Analytical duplicates were included on every tenth sample within analytical runs to ensure repeatability (coefficient of variation <3%).

Crude protein (CP) was quantified by the Kjeldahl method (ISO 5983-1:2005; ISO 1871:2009) using an automated distillation and titration system (TitroLine 5000; SI Analytics, Mainz, Germany). Nitrogen content was multiplied by the standard conversion factor of 6.25 to maintain conventional feed comparability. Additionally, the insect-specific nitrogen-to-protein conversion factor (*Kp* = 4.76–5.60, Janssen et al. [39]) was applied in calculations to quantify the overestimation caused by non-protein nitrogen (NPN) bound in cuticular chitin.

Crude fat (ether extract, EE) was determined gravimetrically using petroleum ether extraction in an automated Soxtec™ 2043 system (FOSS, Hillerød, Denmark; ISO 11085:2015, Procedure A; total extraction time 120 min: 30 min boiling/submersion, 60 min rinsing/percolation, 30 min solvent recovery). Crude ash was determined by incinerating samples in a muffle furnace at 550 ± 25 °C to a constant weight (ISO 5984:2022). Water-soluble carbohydrates (WSCs) were extracted with hot deionized water (80 °C) and measured colorimetrically using the anthrone-sulfuric acid reagent on a UV-Vis spectrophotometer (Genesys 10S, Thermo Fisher Scientific, Waltham, MA, USA) against a D-glucose calibration curve (*R*^2^ > 0.99).

Total phosphorus (P) was determined spectrophotometrically via the molybdovanadate yellow method at 430 nm following sulfuric–perchloric acid wet digestion, calibrated against a standard KH_2_PO_4_ curve. Mineral elements (Ca, K, Na, Mg, Fe, Zn, Mn, Cu) were quantified using flame atomic absorption spectrometry (FAAS; AAnalyst 200, PerkinElmer, Waltham, MA, USA; ISO 6869:2000) after wet acid digestion (HNO_3_ + HCl, 1:3 *v*/*v*) on a hot plate. Limits of detection (LOD) were 0.05 mg/L for Ca, Mg, Na, K, Fe, and Zn, and 0.02 mg/L for Mn and Cu.

### 2.5. Gravimetric Chitin Extraction and Yield Determination

The extracted chitin yield was quantified gravimetrically following an established three-step chemical protocol [19,20]:

1. **Demineralization:** Ground larval biomass was treated with 2 M HCl at a solid-to-liquid ratio of 1:20 (g/mL) at 65 °C for 4 h under continuous magnetic stirring to dissolve mineral incrustations. The acid-insoluble residue was separated by vacuum filtration on Whatman No. 4 filter paper, rinsed with distilled water until a neutral pH, and dried.
2. **Deproteinization:** Demineralized residues were suspended in 2 M NaOH (1:20 g/mL) under reflux at 100–105 °C for 8 h at 500 rpm to hydrolyze structural proteins. The alkali-insoluble chitin residue was collected by filtration, washed repeatedly with distilled water to neutrality, and dried at 60 °C.
3. **Decolorization:** The crude residue was bleached with 1% (*w*/*v*) H_2_O_2_ at 70 °C for 1 h to remove melanin pigments. The bleached chitin was washed with deionized water to neutrality and dried at 60 °C to a constant weight.

The extracted chitin yield was calculated on a larval dry matter basis as

Chitin Yield (%) = (*m_chitin_*/*m_initial_*) × 100 (1)

where *m_chitin_* is the mass of dry extracted chitin residue (g) and *m_initial_* is the initial dry mass of larval biomass (g). Because spectroscopic verification (FTIR) and residual ash analyses were not performed on the isolated fractions, values strictly denote gravimetric yields.

### 2.6. Statistical Analysis

Data were analyzed using IBM SPSS Statistics (Version 28.0; IBM Corp., Armonk, NY, USA). The experimental unit was the rearing container (*n* = 3 independent biological replicates per dietary treatment across rearing cycles, total *N* = 12). Normality of distributions and homogeneity of variances were verified. Differences among dietary treatments were evaluated using one-way analysis of variance (ANOVA). Post hoc pairwise comparisons were performed using Tukey’s honest significant difference (HSD) test. Statistical significance was declared at *p* < 0.05, and exact *p*-values are reported. Results are expressed on a dry matter (DM) basis as mean ± standard deviation (SD).

## 3. Results

### 3.1. Proximate Macronutrients, Ash Content, and Chitin Yield

Dietary formulation significantly affected larval crude protein (*F*_3,8_ = 4.72, *p* = 0.038), crude ash (*F*_3,8_ = 32.14, *p* < 0.001), and the extracted chitin yield (*F*_3,8_ = 14.88, *p* = 0.004; Table 2). In contrast, crude fat (*F*_3,8_ = 0.38, *p* = 0.774) and water-soluble carbohydrates (*F*_3,8_ = 1.15, *p* = 0.388) did not differ significantly among the dietary treatments.

The crude protein content was highest in larvae reared on the 100% wheat bran Control (50.85 ± 3.23% DM). Inclusion of SBP resulted in a significant reduction in crude protein to 44.66 ± 2.88% DM in Option 1, 44.36 ± 3.12% DM in Option 2, and 45.25 ± 0.97% DM in Option 3 (*p* < 0.05 vs. Control). No significant differences were observed among the three SBP-containing diets (*p* > 0.05). When adjusted for non-protein nitrogen using an insect-specific factor (*Kp* = 5.00), the estimated true protein was 40.68% in Control and 35.49–36.20% in SBP treatments, indicating an approximate 12% overestimation when using *N* × 6.25.

The crude fat content remained statistically uniform across diets, ranging from 32.18 ± 8.62% DM in Option 1 to 36.32 ± 5.54% DM in Option 2 (*p* = 0.774). Water-soluble carbohydrate concentrations were consistently low across all treatments (1.80–2.20% DM, *p* = 0.388).

The crude ash content increased markedly with SBP inclusion (*p* < 0.001). Compared to the Control (5.75 ± 0.52% DM), the ash content more than doubled in Option 1 (13.68 ± 0.82% DM) and Option 2 (12.35 ± 0.91% DM), with no significant difference between Options 1 and 2 (*p* = 0.551). In Option 3 (molasses-supplemented), the ash content reached 10.59 ± 2.31% DM, which was significantly lower than Option 1 (*p* = 0.046), but remained significantly higher than the Control (*p* = 0.003).

The extracted chitin yield showed significant dietary responses (*p* = 0.004; Table 2). The highest yields were recovered from Option 2 (7.82 ± 0.18% DM) and Option 1 (7.75 ± 0.22% DM), which did not differ significantly from one another (*p* = 0.941), but were both significantly higher than the Control (7.34 ± 0.15% DM; *p* = 0.032 and *p* = 0.048, respectively). Option 3 yielded the lowest chitin content (7.14 ± 0.12% DM), which was significantly lower than both 40% SBP diets (*p* = 0.005 vs. Option 1; *p* = 0.003 vs. Option 2), but statistically comparable to the Control (*p* = 0.457).

### 3.2. Larval Mineral Profile

The substrate formulation significantly influenced larval concentrations of Ca (*F*_3,8_ = 11.24, *p* = 0.003), P (*F*_3,8_ = 4.88, *p* = 0.032), Fe (*F*_3,8_ = 5.61, *p* = 0.024), and Mn (*F*_3,8_ = 4.41, *p* = 0.041; Table 3). Potassium (*p* = 0.995), sodium (*p* = 0.685), magnesium (*p* = 0.863), zinc (*p* = 0.985), and copper (*p* = 0.869) showed no significant differences among treatments.

The larval calcium content increased more than threefold on SBP diets (*p* = 0.003). Whole-body Ca rose from 2.43 ± 1.39% DM in the Control to 8.06 ± 3.69% DM in Option 1, 8.84 ± 4.75% DM in Option 2, and 7.46 ± 3.29% DM in Option 3. Tukey’s test confirmed that Options 1, 2, and 3 were significantly higher than the Control (*p* < 0.05), with no significant differences among the three SBP diets (*p* > 0.05).

Total phosphorus differed significantly (*p* = 0.032). Phosphorus was significantly higher in Option 1 (1.27 ± 0.08% DM) compared to the Control (0.99 ± 0.03% DM; *p* = 0.018). In Options 2 (1.10 ± 0.08% DM) and 3 (1.12 ± 0.21% DM), phosphorus concentrations were intermediate and did not differ significantly from either the Control or Option 1 (*p* > 0.05). Due to the substantial increase in Ca, the whole-body larval Ca:P ratio expanded from 2.45:1 in the Control to 6.35:1 in Option 1, 8.04:1 in Option 2, and 6.66:1 in Option 3.

Potassium (1.56–1.68% DM), sodium (0.67–0.90% DM), and magnesium (0.59–0.67% DM) showed no statistically significant dietary variation (*p* > 0.05), although sodium showed a non-significant numerical increase in the molasses group (Option 3: 0.90 ± 0.40% DM).

Among microelements, iron was significantly higher in Option 1 (286.9 ± 71.5 mg/kg) than in the Control (168.9 ± 34.4 mg/kg; *p* = 0.024), while Options 2 (228.0 ± 28.5 mg/kg) and 3 (225.3 ± 82.9 mg/kg) exhibited intermediate levels (*p* > 0.05 vs. Control). Manganese was significantly elevated in all SBP diets, reaching 700.6 ± 99.6 mg/kg in Option 2 vs. 589.9 ± 71.7 mg/kg in the Control (*p* = 0.041). Zinc (208.4–234.1 mg/kg; *p* = 0.985) and copper (18.7–25.4 mg/kg; *p* = 0.869) remained statistically comparable across all groups.

## 4. Discussion

### 4.1. Crude Protein Content and NPN Conversion

The larval crude protein content decreased significantly from 50.85% DM in the 100% wheat bran Control to 44.36–45.25% DM on SBP diets. This reduction is primarily explained by dietary nitrogen dilution: raw wheat bran typically contains 15.5–16.8% CP, whereas SBP provides only 8.2–9.8% CP (Table S1) [13,28]. Partially replacing wheat bran with SBP reduced the overall dietary protein density, limiting the substrate amino acid pool available for somatic protein accretion. These findings agree with Barragan-Fonseca et al. [12] and Meneguz et al. [40], who demonstrated that the *H. illucens* protein content is sensitive to dietary protein-to-carbohydrate ratios.

Neither *Trichoderma* inoculation nor molasses addition reversed this decline. In Option 2, single-dose fungal inoculation without a prior solid-state incubation period was insufficient to significantly augment microbial protein biomass. In Option 3, molasses provided fermentable sucrose but virtually no digestible protein, yielding a comparable protein dilution.

Importantly, our values were determined via the conventional Kjeldahl factor of *N* × 6.25. As demonstrated by Janssen et al. [39], non-protein nitrogen (NPN) present in cuticular chitin leads to an overestimation of true protein by approximately 11% to 24%. When adopting an insect-specific factor (*Kp* = 5.00), true protein levels are estimated at 40.68% in the Control and 35.49–36.20% in SBP diets. Feed formulators should account for this NPN fraction when substituting conventional protein ingredients with SBP-reared insect meal.

### 4.2. Lipogenesis and Carbohydrate Utilization

In contrast to crude protein, the larval crude fat content remained stable (32.18–36.32% DM) across all treatments, despite the very low lipid concentration naturally found in SBP (<1.4% DM; Table S1). This demonstrates the robust metabolic capacity of *H. illucens* for *de novo* lipogenesis, converting structural and non-structural carbohydrates into body fat pools [15,16,40]. Microbial fermentation in the larval gut degrades digestible fiber components into volatile fatty acids (VFAs), primarily acetate, which enter the cytosolic acetyl-CoA pool to drive fatty acid synthesis [10,11]. The resulting fat is predominantly characterized by saturated medium-chain fatty acids, especially lauric acid (C12:0) [15,40]. Water-soluble carbohydrates remained uniformly low in larval tissue (1.80–2.20% DM), consistent with the active homeostatic conversion of circulating hexoses into glycogen or triacylglycerols [16].

### 4.3. Crude Ash Dynamics, Gastrointestinal Carryover, and Ca:P Implications

A prominent outcome of SBP feeding was the marked increase in crude ash content, rising from 5.75% DM in the Control to 10.59–13.68% DM on SBP diets, driven largely by calcium accumulation (7.46–8.84% DM vs. 2.43% in Control). In commercial sugar beet extraction, lime (Ca(OH)_2_) and CO_2_ are introduced during juice purification, precipitating calcium carbonate directly onto the exhausted beet pulp [13,27]. Consequently, SBP carries an inherently high calcium concentration (1.10–1.80% DM; Table S1) compared to cereal grains (0.12% DM).

An essential methodological consideration is that larvae in this trial were harvested without a 24 h fasting period, reflecting practical whole-larva meal production. Therefore, the analyzed ash, Ca, and Mn concentrations reflect the total elemental mass of whole larvae, including both physiological tissue deposition and transitory digesta within the larval gastrointestinal tract. While *H. illucens* larvae possess an innate capacity to sequester calcium carbonate into their cuticular exoskeleton [7,8], a measurable fraction of the recorded calcium in SBP-fed larvae represents undigested fibrous bolus retained in the lumen.

Total phosphorus concentrations ranged between 0.99% and 1.27% DM, with Option 1 displaying a modest but statistically significant elevation compared to the Control (*p* = 0.018). The resulting whole-larva Ca:P ratio expanded dramatically from 2.45:1 in the Control to 6.35:1–8.04:1 in SBP diets. In non-ruminant animal nutrition (poultry and swine), optimal dietary Ca:P ratios generally range between 1.2:1 and 2.0:1 [23,41]. Elevated dietary Ca:P ratios can impair animal performance by forming insoluble calcium-phosphate precipitates in the intestinal lumen, reducing the bioavailability of both phosphorus and essential trace elements [41]. Recent work by Cattaneo et al. [41] highlighted that while high-calcium *H. illucens* meal can serve as an exceptional calcium source for laying hens, maintaining the eggshell quality without adverse effects, its incorporation into broiler or piglet diets requires careful formulation and balancing with inorganic monocalcium/dicalcium phosphate or phytase supplementation.

### 4.4. Trace Element Retention and Mineral Interactions

Trace element responses revealed element-specific patterns. Manganese was significantly elevated in all SBP diets (661.2–700.6 mg/kg vs. 589.9 mg/kg in Control, *p* = 0.041), and iron increased in Option 1 (286.9 mg/kg vs. 168.9 mg/kg, *p* = 0.024). Sugar beet roots accumulate manganese and iron from agricultural soils [13], and these elements were carried over into the larval biomass.

Conversely, zinc (208.4–234.1 mg/kg) and copper (18.7–25.4 mg/kg) remained statistically unchanged across treatments (*p* > 0.05). In animal digestive tracts, high calcium concentrations often competitively antagonize zinc and copper absorption through shared mucosal transport pathways [7,23]. The preservation of zinc and copper retention in SBP-fed larvae suggests effective physiological homeostasis. In Option 2, *Trichoderma* secretion of organic acids and low-molecular-weight siderophores may have facilitated micro-environmental acidification and natural chelation, maintaining trace element solubility [31]. Magnesium also remained stable (0.59–0.67% DM), confirming tight metabolic regulation in dipteran tissues [17].

### 4.5. Gravimetric Chitin Yield

The gravimetric chitin yield was significantly higher in larvae reared on 40% SBP diets (7.75% DM in Option 1; 7.82% DM in Option 2) compared to the Control (7.34% DM; *p* < 0.05). Chitin synthesis is tightly linked to cuticular development [18,20]. Fibrous diets with a higher structural carbohydrate content and lower digestible protein may increase cuticular surface area-to-volume ratios relative to somatic fat accumulation, or promote thicker procuticle formation [20,21].

Option 1 and Option 2 did not differ significantly from each other, indicating that *Trichoderma* inoculation did not enhance the chitin yield beyond raw SBP. Single-dose fungal inoculation without a prolonged pre-fermentation phase likely limited fungal biomass proliferation (fungal cell walls contain chitin), thereby having a negligible impact on the total yield.

Notably, Option 3 yielded the lowest chitin percentage (7.14 ± 0.12% DM), which was significantly lower than the 40% SBP diets (*p* < 0.01). Option 3 combined a 10% reduction in SBP (from 40% to 30%) with the addition of 10% molasses. This outcome represents the combined net effect of both formulation changes: reducing the structural fiber fraction while providing highly accessible sucrose shifted metabolic carbon partitioning toward somatic lipid and carbohydrate storage rather than cuticular chitin polymerization [21,30].

From an animal nutrition perspective, moderate chitin levels (6–8% DM) act as prebiotic dietary fiber, supporting beneficial gut microbiota (e.g., *Lactobacillus* and *Bifidobacterium*) and enhancing gut barrier integrity in poultry and swine [23,41]. However, excessive cuticular chitin reduces total tract protein digestibility in young monogastric animals [18,23].

### 4.6. Study Limitations and Future Research Directions

Several methodological limitations should be considered:

1. Absence of Batch-Specific Substrate Analysis: Initial proximate and mineral analyses of the specific raw ingredient batches were not performed. While literature baseline data (Table S1) provide an informative nutritional frame, direct mass-balance calculations, bioaccumulation factors (BAFs), and bioconversion efficiencies could not be determined.
2. Gut Evacuation: Larvae were processed without a 24 h pre-harvest fasting period. Although this accurately represents commercial whole-larva processing, it does not allow complete separation between assimilated tissue minerals and luminal digesta residue.
3. Option 3 Confounding: Option 3 simultaneously reduced SBP and added molasses; consequently, the independent metabolic effect of molasses cannot be statistically isolated.
4. Chitin Characterization: Chitin was determined gravimetrically; structural spectroscopy (FTIR) and residual ash/protein verification were not conducted to verify polymer purity.
5. Protein and Lipid Profiling: Detailed amino acid and fatty acid profiles were not determined, precluding direct evaluation of essential amino acid scores or fatty acid saturation indices.

Future research should utilize factorial dietary designs, include 24 h gut clearance protocols alongside whole-larva processing, and perform spectroscopic polymer validation to fully elucidate the bioconversion mechanisms of *H. illucens* on agro-industrial side-streams.

## 5. Conclusions

Incorporating 40% sugar beet pulp (SBP) into *Hermetia illucens* diets significantly reduced the larval crude protein content from 50.85% DM to 44.36–45.25% DM, reflecting dietary protein dilution. However, the larval crude fat content remained stable at 32.18–36.32% DM, demonstrating efficient *de novo* lipogenesis on high-fiber diets.

SBP inclusion markedly altered the mineral profile of whole-larval biomass, more than doubling the crude ash content (10.59–13.68% DM vs. 5.75% DM) and increasing whole-body calcium (7.46–8.84% DM vs. 2.43% DM) and manganese concentrations. This widened the larval Ca:P ratio from 2.45:1 to 8.04:1. Because larvae were harvested without a pre-harvest fasting period, these elevated mineral levels reflect the whole-body commercial product, combining cuticular mineral sequestration with gastrointestinal bolus carryover.

The extracted gravimetric chitin yield was moderately elevated on 40% SBP diets (7.75–7.82% DM) compared to the Control (7.34% DM), whereas replacing 10% SBP with molasses in Option 3 produced the lowest chitin yield (7.14% DM), reflecting a net shift toward somatic storage. *Trichoderma* sp. inoculation without extended pre-fermentation produced no significant differences over SBP alone.

For animal feed formulators, whole-larval biomass reared on SBP-containing substrates represents an energy- and calcium-dense feed ingredient. It is well-suited as a natural calcium and protein supplement for laying poultry, but requires dietary Ca:P rebalancing and supplemental phosphorus when formulated for young swine or broiler chickens.

## Figures and Tables

**Table 1:** Formulation of experimental diets evaluated for *Hermetia illucens* larvae.

Treatment	Wheat Bran (% DM)	Sugar Beet Pulp (% DM)	Molasses (% DM)	Additive	Wheat Bran (% As-Fed)	SBP (% As-Fed)	Molasses (% As-Fed)
Control	100	0	0	None	34.0	0	0
Option 1	60	40	0	None	20.4	54.5	0
Option 2	60	40	0	*Trichoderma* sp. (1 × 10^6^ CFU/g)	20.4	54.5	0
Option 3	60	30	10	None	20.4	40.9	4.5

Note: All diets were adjusted to a 70% final moisture content with distilled water prior to larval introduction. As-fed values reflect typical raw ingredient moisture contents (wheat bran ~12%, wet pressed SBP ~78%, beet molasses ~25%).

**Table 2:** Proximate macronutrient composition, water-soluble carbohydrates, crude ash, and gravimetric chitin yield of *Hermetia illucens* larval biomass on dry matter (DM) basis across dietary treatments (mean ± SD, *n* = 3).

Parameter (% DM)	Control (100% Bran)	Option 1 (40% SBP)	Option 2 (40% SBP + *Trichoderma*)	Option 3 (30% SBP + 10% Molasses)	*p*-Value
Crude Protein (CP)	50.85 ± 3.23 ^a^	44.66 ± 2.88 ^b^	44.36 ± 3.12 ^b^	45.25 ± 0.97 ^b^	0.038
Crude Fat (EE)	33.42 ± 2.52 ^a^	32.18 ± 8.62 ^a^	36.32 ± 5.54 ^a^	35.57 ± 0.63 ^a^	0.774
Water-Soluble Carbohydrates (WSCs)	2.20 ± 0.27 ^a^	1.80 ± 0.27 ^a^	1.82 ± 0.37 ^a^	1.81 ± 0.40 ^a^	0.388
Crude Ash	5.75 ± 0.52 ^c^	13.68 ± 0.82 ^a^	12.35 ± 0.91 ^a^	10.59 ± 2.31 ^b^	<0.001
Extracted Chitin Yield	7.34 ± 0.15 ^b^	7.75 ± 0.22 ^a^	7.82 ± 0.18 ^a^	7.14 ± 0.12 ^c^	0.004

Note: Values represent the mean ± SD of three independent biological rearing containers (*n* = 3). Means within a row sharing different superscript letters (^a,b,c^) differ significantly according to Tukey’s HSD test (*p* < 0.05).

**Table 3:** Macroelement (%) and microelement (mg/kg) mineral concentrations in *Hermetia illucens* larval biomass on a dry matter (DM) basis across dietary treatments (Mean ± SD, *n* = 3).

Mineral Element	Control (100% Bran)	Option 1 (40% SBP)	Option 2 (40% SBP + *Trichoderma*)	Option 3 (30% SBP + 10% Molasses)	*p*-Value
Calcium (Ca), %	2.43 ± 1.39 ᵇ	8.06 ± 3.69 ᵃᵇ	8.84 ± 4.75 ᵃ	7.46 ± 3.29 ᵃᵇ	0.003
Phosphorus (P), %	0.99 ± 0.03 ᵇ	1.27 ± 0.08 ᵃ	1.10 ± 0.08 ᵃᵇ	1.12 ± 0.21 ᵃᵇ	0.032
Potassium (K), %	1.56 ± 0.56 ᵃ	1.59 ± 0.65 ᵃ	1.68 ± 0.76 ᵃ	1.58 ± 0.64 ᵃ	0.995
Sodium (Na), %	0.67 ± 0.10 ᵃ	0.69 ± 0.18 ᵃ	0.74 ± 0.35 ᵃ	0.90 ± 0.40 ᵃ	0.685
Magnesium (Mg), %	0.63 ± 0.09 ᵃ	0.64 ± 0.10 ᵃ	0.67 ± 0.19 ᵃ	0.59 ± 0.13 ᵃ	0.863
Iron (Fe), mg/kg	168.9 ± 34.4 ᵇ	286.9 ± 71.5 ᵃ	228.0 ± 28.5 ᵃᵇ	225.3 ± 82.9 ᵃᵇ	0.024
Zinc (Zn), mg/kg	234.1 ± 124.5 ᵃ	218.6 ± 82.2 ᵃ	208.4 ± 113.8 ᵃ	217.9 ± 99.2 ᵃ	0.985
Manganese (Mn), mg/kg	589.9 ± 71.7 ᵇ	661.2 ± 73.3 ᵃᵇ	700.6 ± 99.6 ᵃ	678.4 ± 44.5 ᵃᵇ	0.041
Copper (Cu), mg/kg	25.4 ± 17.1 ᵃ	23.2 ± 15.1 ᵃ	19.7 ± 8.7 ᵃ	18.7 ± 10.4 ᵃ	0.869
Calculated Ca:P ratio	2.45:1	6.35:1	8.04:1	6.66:1	—

Note: Values represent the mean ± SD (*n* = 3). Means within a row sharing different superscript letters (^a,b^) differ significantly according to Tukey’s HSD test (*p* < 0.05). Exact *p*-values derive from one-way ANOVA.

## Data Availability

The data presented in this study are available on request from the corresponding author. The authors gratefully acknowledge the staff of the Chemical Research Laboratory at the Institute of Agriculture, Lithuanian Research Centre for Agriculture and Forestry (LAMMC) for their assistance and technical support during the chemical analyses.

## Supplementary Materials

The following supporting information can be downloaded at https://www.mdpi.com/article/10.3390/ani16213380/s1, Table S1: Representative nutritional baseline composition of raw dietary ingredients (literature values, dry matter basis).
