# Supplementary material for: Impact of Sugar Beet Pulp-Based Diets on the Chemical Composition, Mineral Profile, and Chitin Yield of Black Soldier Fly (Hermetia illucens L.) Larvae
Source: Animals (Basel). 2026 Oct 27;16(21):3380. doi: 10.3390/ani16213380 (PMC13762114; doi:10.3390/ani16213380)
Supplement: Supplementary file 1 [file animals-16-03380-s001.zip › animals-4543656-supplementary.pdf]

Table S1. Representative nutritional baseline composition of raw dietary ingredients (literature values, dry matter basis).

Component	Wheat Bran [40]	Sugar Beet Pulp [22,24]	Beet Molasses [41]
Dry Matter (DM), %	87.0–89.0	20.0–24.0	74.0–78.0
Crude Protein (CP), % DM	15.5–16.8	8.2–9.8	8.5–10.2
Crude Fat (EE), % DM	3.8–4.5	0.8–1.4	0.1–0.3
Crude Ash, % DM	5.2–6.0	6.8–9.5	8.5–11.0
Neutral Detergent Fiber (NDF), % DM	40.0–45.0	45.0–52.0	0.0
Calcium (Ca), % DM	0.12–0.15	1.10–1.80	0.20–0.35
Phosphorus (P), % DM	1.05–1.18	0.08–0.12	0.02–0.05
Potassium (K), % DM	1.10–1.35	0.70–1.10	3.50–5.20
